# High titers of neutralizing SARS-CoV-2 antibodies six months after symptom onset are associated with increased severity in COVID-19 hospitalized patients

**DOI:** 10.1186/s12985-023-01974-8

**Published:** 2023-01-25

**Authors:** Adin Sejdic, Anders Frische, Charlotte Sværke Jørgensen, Lasse Dam Rasmussen, Ramona Trebbien, Arnold Dungu, Jon G. Holler, Sisse Rye Ostrowski, Robert Eriksson, Christian Søborg, Thyge L. Nielsen, Thea K. Fischer, Birgitte Lindegaard, Kristina Træholt Franck, Zitta Barrella Harboe

**Affiliations:** 1 Department of Pulmonary and Infectious Diseases, Copenhagen University Hospital, North Zealand, Hillerød, Denmark; 2 Statens Serum Institut, Copenhagen, Denmark; 3 Department of Clinical Medicine, Faculty of Health and Medical Sciences, University of Copenhagen, Copenhagen, Denmark; 4 Department of Clinical Immunology, Copenhagen University Hospital – Rigshospitalet, Copenhagen, Denmark; 5 Department of Clinical Research, Copenhagen University Hospital – North Zealand, Copenhagen, Denmark; 6 Department of Infectious Diseases, Karolinska Institutet, Solna, Sweden; 7 Department of Public Health, Faculty of Health and Medical Sciences, University of Copenhagen, Copenhagen, Denmark

**Keywords:** COVID-19, Neutralizing antibodies, Viral culturing, Viral load, Disease severity

## Abstract

**Background:**

Viral shedding and neutralizing antibody (NAb) dynamics among patients hospitalized with severe coronavirus disease 2019 (COVID-19) and immune correlates of protection have been key questions throughout the pandemic. We investigated the duration of reverse transcriptase-polymerase chain reaction (RT-PCR) positivity, infectious viral shedding and NAb titers as well as the association between NAb titers and disease severity in hospitalized COVID-19 patients in Denmark 2020–2021.

**Materials and methods:**

Prospective single-center observational cohort study of 47 hospitalized COVID-19 patients. Oropharyngeal swabs were collected at eight time points during the initial 30 days of inclusion. Serum samples were collected after a median time of 7 (IQR 5 – 10), 37 (IQR 35 – 38), 97 (IQR 95 – 100), and 187 (IQR 185 – 190) days after symptom onset. NAb titers were determined by an in-house live virus microneutralization assay. Viral culturing was performed in Vero E6 cells.

**Results:**

Patients with high disease severity had higher mean log_2_ NAb titers at day 37 (1.58, 95% CI [0.34 –2.81]), 97 (2.07, 95% CI [0.53–3.62]) and 187 (2.49, 95% CI [0.20– 4.78]) after symptom onset, compared to patients with low disease severity. Peak viral load (0.072, 95% CI [− 0.627 – 0.728]), expressed as log_10_ SARS-CoV-2 copies/ml, was not associated with disease severity. Virus cultivation attempts were unsuccessful in almost all (60/61) oropharyngeal samples collected shortly after hospital admission.

**Conclusions:**

We document an association between high disease severity and high mean NAb titers at days 37, 97 and 187 after symptom onset. However, peak viral load during admission was not associated with disease severity.

*Trial registration*. The study is registered at https://clinicaltrials.gov/ (NCT05274373).

**Supplementary Information:**

The online version contains supplementary material available at 10.1186/s12985-023-01974-8.

## Background

Coronavirus disease 2019 (COVID-19) is a respiratory illness caused by the β-coronavirus severe acute respiratory syndrome coronavirus 2 (SARS-CoV-2) and is the cause of the pandemic [1]. Humoral immunity is vital to combat and protect from SARS-CoV-2 infection [2]. Therefore, understanding clinical factors affecting humoral protection over time is essential to understanding the disease caused by SARS-CoV-2.

Antibodies (Ab) against the receptor-binding domain (RBD) of the SARS-CoV-2 spike protein are crucial for developing immunological protection [3]. Several factors may influence humoral responses to SARS-CoV-2 infection and vaccination, including increasing age, male sex and immunosuppression [4–8]. However, how disease severity influences humoral responses, such as neutralizing antibodies (NAb) production, is not fully understood [2]. A positive association between NAb titers, using different laboratory assays, and disease severity has been well described for up to 90 days after symptom onset [9–14]. However, the effect of disease severity on NAb titers beyond 90 days after symptom onset is currently lacking.

An extensive review concluded that the association between viral load and disease severity is inconsistent [15]. Therefore, assessing viral shedding and clinical characteristics affecting it is essential to identify and isolate infectious patients correctly and  to further assess the inconsistent relationship between viral load, disease severity and humoral responses over time.

We conducted a prospective cohort study to evaluate humoral responses and live viral shedding in patients hospitalized with COVID-19. In addition, we explored whether clinical characteristics, such as disease severity, could affect NAb titers for up to 180 days and viral shedding for up to 30 days after study inclusion.

## Materials and methods

### Study design and population

Patients 18 years or older hospitalized at Copenhagen University Hospital—North Zealand, Denmark, between May 24, 2020, and May 5, 2021, were screened for COVID-19 at admission by routine collection and analysis of oropharyngeal swabs or tracheal aspirate samples. The swabs and aspirates were locally analyzed in a diagnostic reverse transcriptase-polymerase chain reaction (RT-PCR) assay as part of the hospital routine at admission. Inclusion criteria for the study were: (1) positive SARS-CoV-2 respiratory tract specimen (virological criteria) within 48 h of study inclusion, (2) consolidations on chest X-ray described by a radiologist or physician (radiological criteria) and (3) the presence of one or more of the following: temperature ≥ 38.0 °C, new-onset cough, pleuritic chest pain, dyspnea or altered breath sounds on auscultation (clinical criteria). Exclusion criteria were: (1) cognitive impairment prohibiting giving informed consent to participation and (2) by December 14, 2020, and onwards, if the time since symptom onset was more than seven days at the time of inclusion.

### Variables and outcomes

Clinical variables extracted from the patient’s electronic medical records and the definition of immunocompromised status are described in the Additional file 1: appendix. Disease severity was defined based on the maximum required oxygen treatment during the hospitalization. Patients defined as having severe disease received high-flow nasal cannula (HFNC), invasive or non-invasive mechanical ventilation (NIV) treatment during the admission. The remaining patients were defined as having a mild disease.

Primary outcomes were defined as (1) NAb titers on days 0, 30, 90 and 180 and (2) viral load during the initial 30 days of inclusion. The secondary outcome was defined as the number of successful viral culturing attempts during the initial 30 days of inclusion.

### Sample collection

Oropharyngeal swabs were collected using flocked swabs in a universal transportation medium (COPAN Italia S.p.A, Brescia, Italy). Oropharyngeal swabs and serum samples were collected on inclusion (day 0), days 3, 7, 10, 14, 17, 24 and 30 and serum was furthermore collected 90 and 180 days after study inclusion. In addition, a control oropharyngeal SARS-CoV-2 RT-PCR sample for immediate analysis was taken on day 14; if negative, no further oropharyngeal sampling was performed.

### Laboratory analyses

See Additional file 1: appendix for a detailed description of reverse transcriptase-quantitative polymerase chain reaction (RT-qPCR), viral culturing and NAb assay methods.

### RT-qPCR

All collected oropharyngeal samples were stored at − 80 °C. RT-qPCR and an attempt to culture virus from RT-PCR positive samples were performed on all swab samples using in-house analyses. Briefly, the RT-qPCR analysis targeted the SARS-CoV-2 RNA-dependent-RNA-polymerase (RdRp)-helicase gene region and two samples with known viral load were included in each PCR-run for quantification of patient samples [16].

### Viral cultures

SARS-CoV-2 was cultured in African green monkey kidney cells (VERO-E6) with incubation for 3–4 days and daily microscopic inspection for cytopathogenic effect (CPE) in accordance with the in-house procedures. A total of three passages were made before the virus was interpreted as non-replicant. In addition, cells with CPE were tested for SARS-CoV-2 RNA by RT-qPCR.

### SARS-CoV-2 Ab

The presence of specific Ab against SARS-CoV-2 in serum was assessed by determining total-Ab by ELISA according to the manufacturer’s instructions (Wantai, Beijing, China). The Wantai ELISA used was reported to have 96.7% and ≥ 99% sensitivity and specificity, respectively. Detailed methods regarding the Wantai ELISA have been published elsewhere [17].

### Microneutralization assay

The microneutralization assay methods and validation used in this study has been published as a separate paper [18]. Briefly, levels of neutralizing antibodies were determined using a median tissue culture infectious dose (TCID_50_) microneutralization assay with an ELISA readout, further described in the Additional file 1: Appendix. Briefly, the 50% neutralization titers were calculated as the interception between a 4-parameter logistic regression curve fitted optical density values from each serum serial dilution and a 50% cut-off value, calculated from quadruplicate virus and cell control wells included on each plate. The titers were normalized according to a positive control on each assay plate to minimize inter-assay variation [19, 20].

### Statistical analysis

Mann–Whitney U test and Fisher’s exact test were used to compare groups. To present results in relation to symptom onset, the median time from symptom onset to sampling time point was added, as appropriate. A linear mixed-effect model (LME) with an unstructured covariance pattern was used to explore associations between repeated NAb titer measurements (dependent variable) and sample day, disease severity, age, sex, and disease severity/sample day interaction (fixed effects). Patient was used as random effect. The LME NAb model was further used to predict mean NAb titers at median days 7, 37, 97 and 187 from symptom onset. Samples exclusively from non-vaccinated patients at the time of sample collection were used in the NAb LME model. A generalized linear model (GLM) was used to assess the association between peak viral load, age, sex, and disease severity (dependent variable). Missing data analysis was conducted and missing completely at random (MCAR) was concluded for the dependent variable (NAb titer). All statistical analyses were performed in R Statistical Software (version 3.6.1) [21].

## Results

**Fig. 1:**
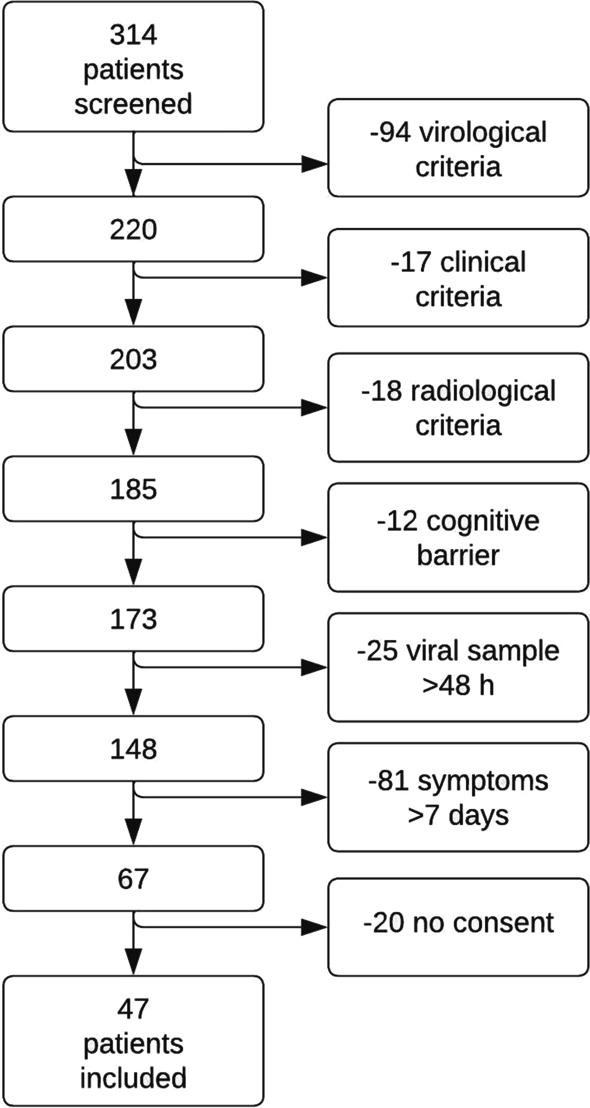
Flowchart describing screening process and patient exclusions

In total, 67 patients were eligible for inclusion, of whom 47 provided informed consent and were enrolled in the study (Fig. 1).

All patients were white Caucasians. None of the included patients had a positive SARS-CoV-2 sample prior to admission, all patients were admitted with primary SARS-CoV-2 infection. A total of 39 patients were included before January 2021, when the wildtype-like variant (formerly referred to as the Wuhan variant) was the dominating circulating variant. Only 8 patients were included January to May 2021 when alpha (B.1.1.7) was the dominant circulating variant in Denmark. Nine patients (19%) had high disease severity during the admission. No patients were known to be diagnosed with a primary or secondary immunodeficiency. No difference in age, sex and number of comorbidities were observed between the two disease severity groups (Table 1). Of seven (15%) patients admitted to the ICU, one (14%) died. In total, two (4%) patients died during admission, and one (2%) died within 180 days after discharge. Of the collected samples on day 180, eight patients (53%) received their first vaccine injection between days 90 and 180 after inclusion. All eight vaccinated patients in the study received the BNT162b2 (Pfizer/BioNTech) vaccine. None of the patients included in this study were vaccinated at the time of admission.

**Table 1:** Patient characteristics of all included patients stratified by disease severity

	Overall, *N* = 47	Disease severity		*p*-value
		Low, *N* = 38	High, *N* = 9	
Baseline characteristics				
Age, years (IQR)	70 (60, 79)	68 (59, 78)	71 (61, 80)	0.53
Male sex, n (%)	31 (66)	23 (61)	8 (89)	0.14
Number of comorbidities				> 0.99
> = 2, *n* (%)	20 (43)	16 (42)	4 (44)	
0–1, *n* (%)	27 (57)	22 (58)	5 (56)	
CCI-score (IQR)	4 (2, 6)	4 (2, 6)	4 (3, 5)	0.81
Immunodeficiency*	0 (0)	0 (0)	0 (0)	
Clinical characteristics				
EWS score at admission (IQR)	5 (3, 7)	5 (2, 6)	6 (5, 7)	0.10
Days since symptom onset at inclusion, days (IQR)	7 (4, 10)	7 (4, 10)	6 (4, 7)	0.24
No assisted respiration, *n* (%)	9 (19)	9 (24)	0 (0)	0.17
Nasal cannula/mask < 5 L oxygen/min, *n* (%)	26 (55)	26 (68)	0 (0)	< 0.001
Mask > = 5 L oxygen/min, *n* (%)	3 (6.5)	3 (7.9)	0 (0)	> 0.99
High-flow oxygen therapy, *n* (%)	4 (8.5)	0 (0%)	4 (44)	< 0.001
Respirator, *n* (%)	3 (6.5)	0 (0)	3 (33)	0.005
NIV, *n* (%)	2 (4.5)	0 (0)	2 (22)	0.033
Admission length, days (IQR)	5 (3, 10)	4 (3, 7)	19 (11, 30)	< 0.001
Death during admission, *n* (%)	2 (4.5)	1 (2.6)	1 (11)	0.35
Death within 180 days, *n* (%)	3 (6.5)	1 (2.6)	2 (22)	0.090

*P*-value was calculated using Mann–Whitney *U* test for numerical variables and Fisher’s exact test for categorical variables

*CCI* Charlson comorbidity index, *EWS* Early warning score, *NIV* Non-invasive ventilation

^*^Immunodeficiency was defined as: the use of (1) corticosteroid treatment exceeding a prednisolone-equivalent dose of 20 mg daily ≥ 14 days at the time of admission, (2) monoclonal antibodies interfering with the immune system, (3) small molecular immunosuppressants, (4) antineoplastic agents, or (5) a primary immunodeficiency diagnosis

### Non-quantitative antibodies and RT-PCR

**Fig. 2:**
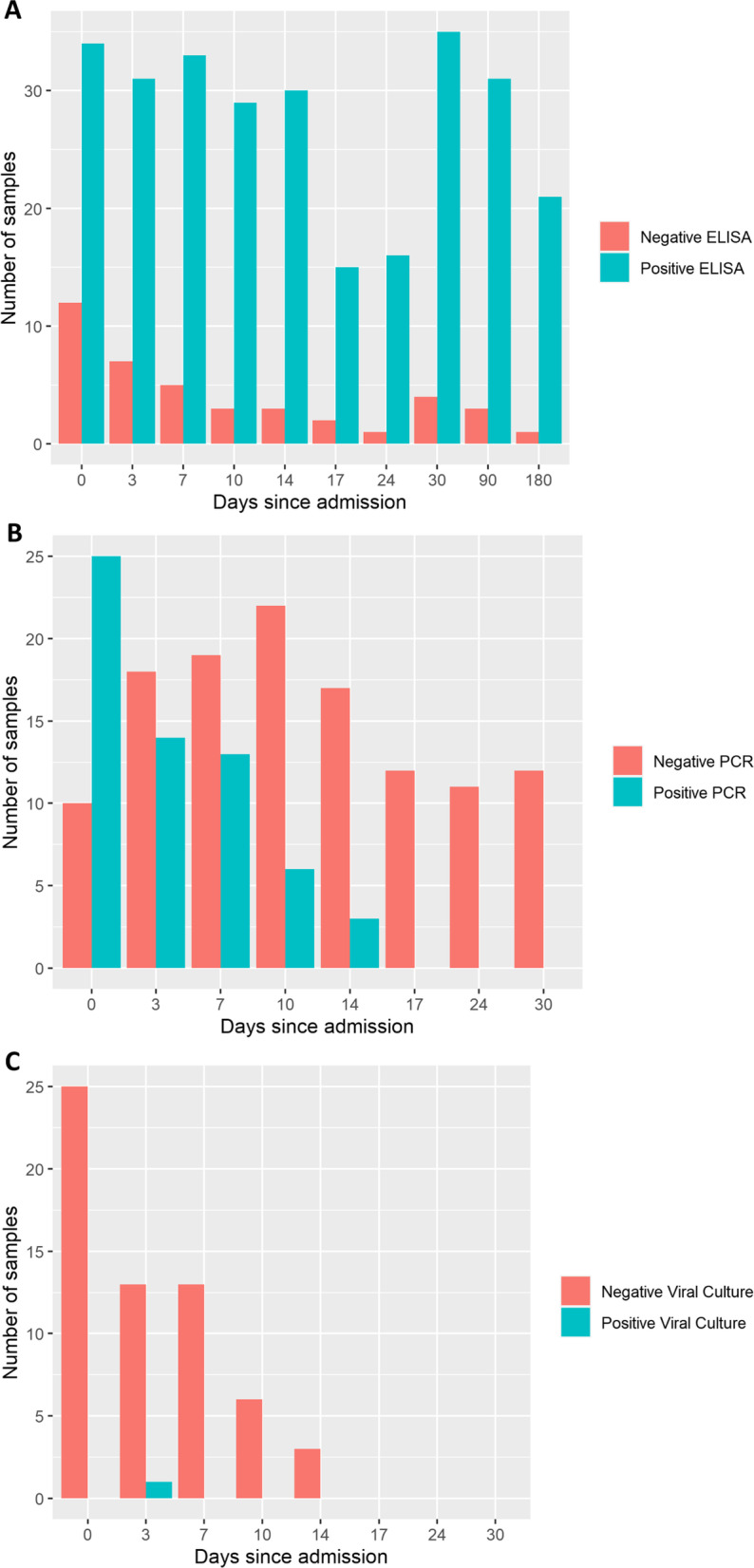
Bar charts describing total number of analyzed samples and the analysis results. **A** Number of analyzed Total Ab ELISA samples. **B** Number of analyzed RT-PCR samples. **C** Number of analyzed viral culturing samples

Non-quantitative total Ab, RT-PCR and viral culturing results are summarized in Fig. 2. Most patients (*n* = 32, 74%) had detectable antibodies upon inclusion. Of the collected Wantai ELISA Ab samples on day 180, one patient (4.5%) did not produce any detectable antibodies. All patients were SARS-CoV-2 RT-PCR negative by day 17 after inclusion.

### Neutralizing antibody titer levels

**Fig. 3:**
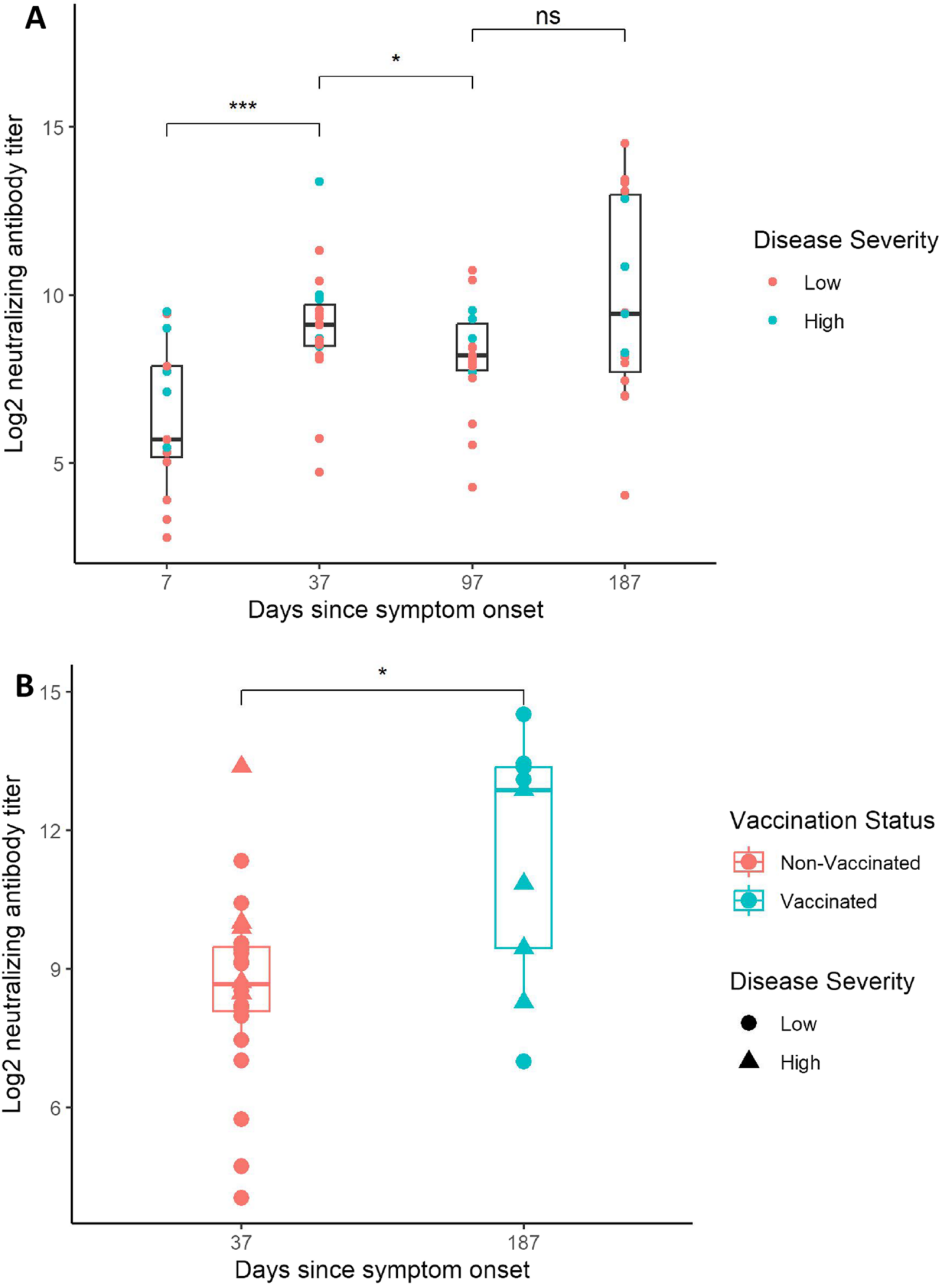
Boxplots describing neutralizing antibody (NAb) titer levels measured at day 0, 30, 90 and 180 since hospital admission. The mean days since symptom onset at admission was seven days and is added to the sampling time points in the figures to describe NAb titers in relation to symptom onset. All titers are presented in log_2_ scale. **A** Boxplots describing log_2_ NAb titers for all measurements taken at each sampling time point. Blue dots represent measurements from patients with high disease severity while red dots represent measurements ﻿from patients with low disease severity. **B** Comparison between vaccine-induced NAb titer responses at day 187 and NAb titer responses following natural infection without prior vaccination at day 37 since symptom onset. The blue boxplot represents measurements from vaccinated patients while the red boxplot represents measurements from non-vaccinated patients. Patients with high disease severity are marked with a triangle while patients with low disease severity are marked as dots. Mann–Whitney U tests were performed to test the null-hypothesis. *** = *P* < 0.001, ** = *P* < 0.01, * = *P* < 0.05, Ns Not significant

**Fig. 4:**
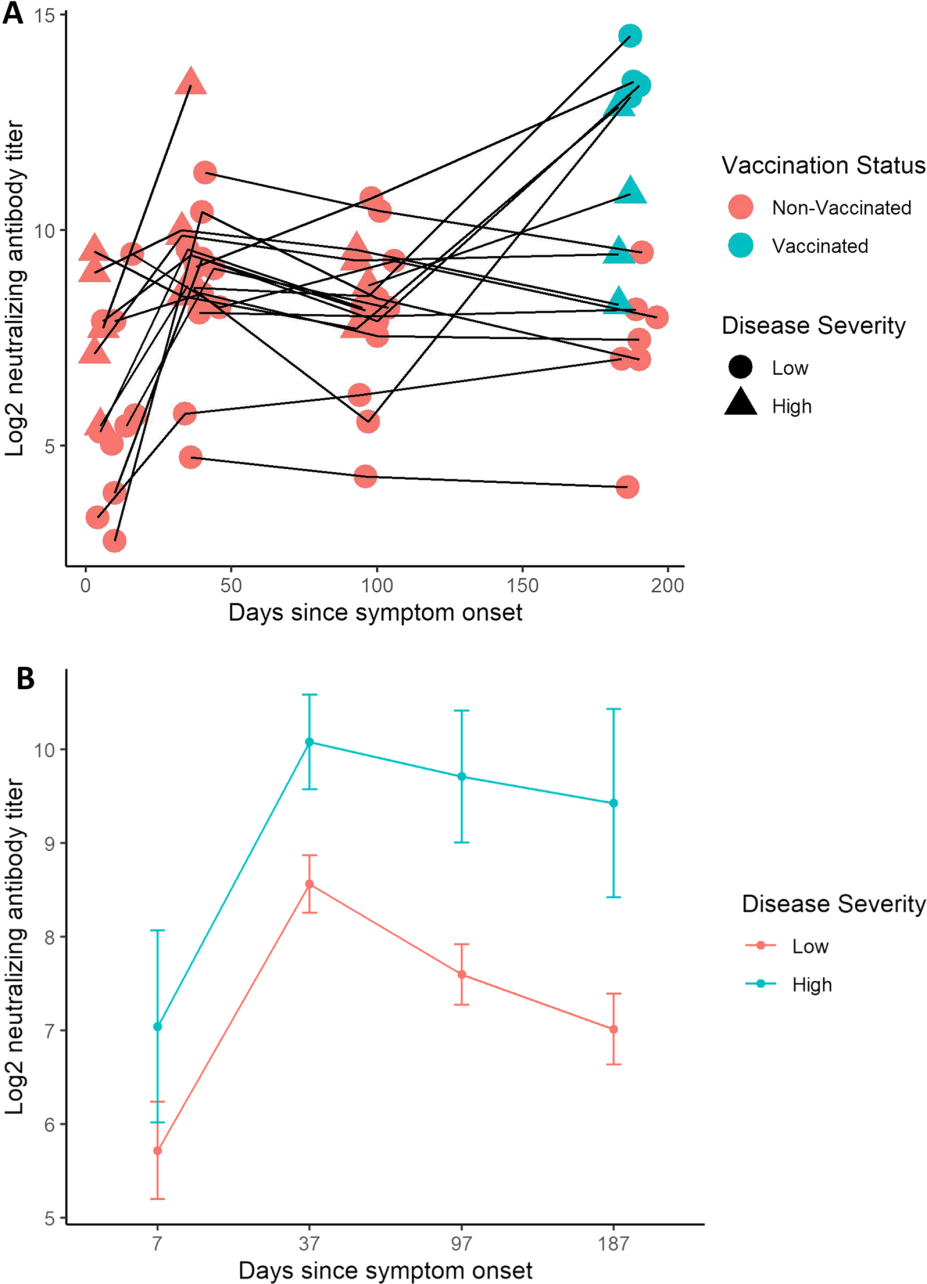
Spaghetti plot with serial neutralizing antibody (NAb) measurements and the corresponding linear mixed-effects (LME) model predictions. NAb titer measurements from the predefined sampling time points at day 0, 30, 90 and 180 after hospital admission were used. The mean days since symptom onset at admission was seven days and is added to the sampling time points in the figures to describe NAb titers in relation to symptom onset. All titers are presented in log_2_ scale. **A** Spaghetti plot showing all serial NAb titer measurements from the predefined sampling time points. Samples from the same patients are marked with connecting black lines. Dots and triangles represent NAb measurement at a specific time point. Vaccination prior to sample collection is marked in blue, while samples from non-vaccinated patients are marked in red. Patients with high disease severity are marked with a triangle, while patients with low disease severity are marked with a dot. **B** LME model predictions with model predictions stratified by disease severity. Samples from vaccinated patients on day 187 were excluded from the model. Standard error is plotted as error bars

Median time from symptom onset to study inclusion was seven days. Serum samples from 15, 19, 18 and 15 patients were analyzed for NAb titers on median days 7 (IQR 5 – 10), 37 (IQR 35 – 38), 97 (IQR 95 – 100), and 187 (IQR 185 – 190) after symptom onset, respectively. The log_2_ NAb titers increased from day 7 to day 37 after symptom onset (*p* < 0.001), and a slight decline between day 37 and day 97 was observed (*p* < 0.05, Fig. 3A). No patients were vaccinated at the time of admission and no patients were vaccinated between admission and day 90. On day 187 since symptom onset, eight (53%) patients were vaccinated before sample collection, resulting in a large variation in NAb titers. Vaccinated patients on day 187 since symptom onset had a higher NAb titer compared to the peak NAb titer for non-vaccinated patients on day 37 (Fig. 3B). Patients with high disease severity had a higher mean log_2_ NAb titer at day 37 (1.58, 95% CI [0.34 –2.81]), 97 (2.07, 95% CI [0.53 –3.62]) and 187 (2.49, 95% CI [0.20– 4.78]) after symptom onset, compared to patients with low disease severity. Model predictions of the mean NAb titer at days 7, 37, 97 and 187 since symptom onset are presented in Fig. 4B. No significant difference in mean NAb titer between high and low disease severity was observed on day 7 (1.41, 95% CI [− 1.08 – 3.90]) since symptom onset. No association between log_2_ NAb titers and age (0.02, 95% CI [− 0.02 – 0.07]) or sex (0.07, 95% CI [− 1.09 – 1.24]) was observed in the model.

### RT-qPCR and viral culturing

**Fig. 5:**
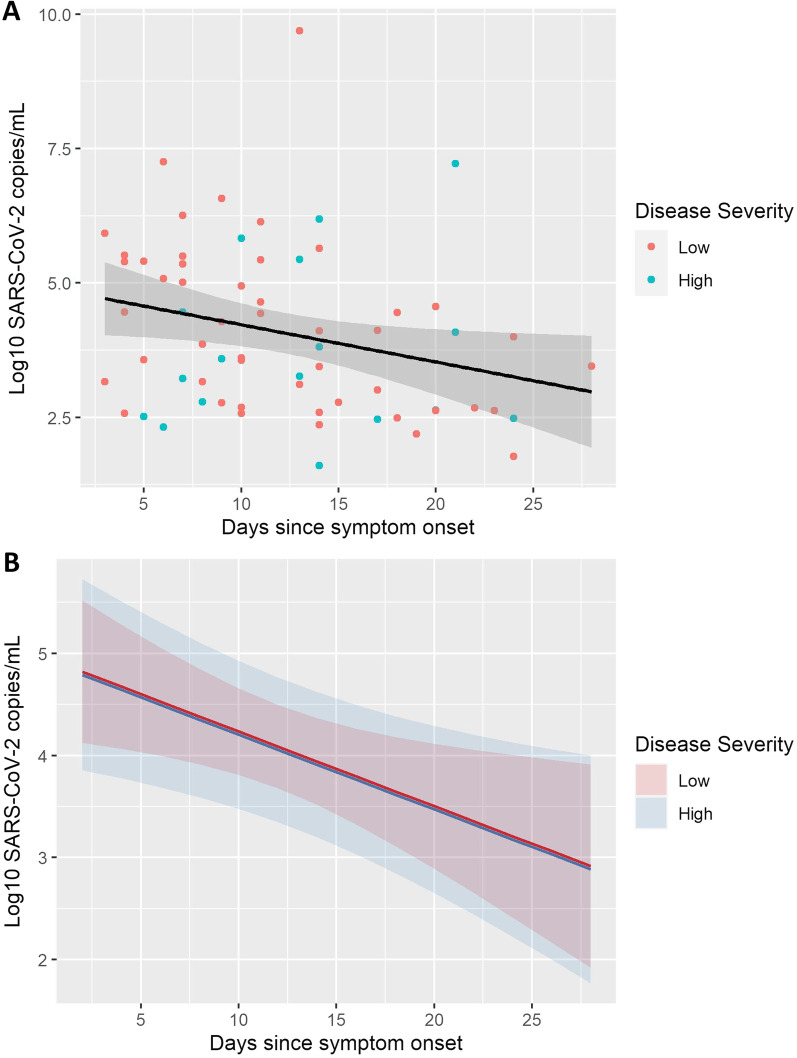
Scatter plot with a linear regression line describing log_10_ viral loads (log_10_ SARS-CoV-2 copies/mL) and a LME model predicting viral loads based on disease severity during the first 30 days of admission. Negative SARS-CoV-2 RT-PCR samples are not presented. All of the available positive samples were collected on days 0, 3, 7, 10 or 14 after hospital admission. The results are plotted in relation to symptom onset. **A** Scatter plot describing viral loads up to 30 days after symptom onset. Regression line is marked in black. Estimated decline in log_10_ viral load was –0.07 per day (-0.07, 95% CI [− 0.13–− 0.02]. Model used to calculate the decline in viral load over time was created with viral load as outcome and days since symptom onset, age, sex, and disease severity as predictors. Standard error is highlighted in grey around the black regression line. **B** LME model demonstrating the effect of disease severity on viral loads over time. Disease severity was not associated with an increase or a decrease in viral loads (− 0.03, 95% CI [− 0.80 – 0.76], *p* = 0.94). Standard error is highlighted in red (low disease severity) or blue (high disease severity) in the background depending on disease severity. Same outcome and predictors as in 5A were used in 5B

A total of 25, 14, 13, 6 and 5 RT-PCR tests were analyzed as positive on days 0, 3, 7, 10 and 14 since inclusion, respectively. The patients had a steady, but non-significant daily decrease in log_10_ SARS-CoV-2 copies/ml during the admission (-0.03, 95% CI [-0.11 –0.05], Fig. 5). Median viral load at day 0 (inclusion) was 4.45 log_10_ copies/ml (IQR 3.16–5.49). Peak viral load (0.072, 95% CI [− 0.627 – 0.728]), expressed as log_10_ SARS-CoV-2 copies/ml, age (0.01, 95% CI [− 0.06 – 0.09]) and sex (1.11, 95% CI [− 1.53 – 4.47]) were not associated with disease severity. The effect of disease severity on viral loads ﻿are depicted in Fig. 5B. Viral culturing was attempted on all 61 positive RT-PCR samples, of which only one (1.5%) successful attempt was observed (Fig. 2).

## Discussion

The major finding of this study was that high disease severity during admission was associated with higher NAb titers for up to 6 months after symptom onset in patients hospitalized due to COVID-19. Also, viral culturing from oropharyngeal swabs taken at hospital admission was difficult due to a long time between symptom onset and hospital admission. Finally, no association between peak viral load during admission and disease severity was observed.

Few studies have addressed whether NAb titers remain higher over time in patients with severe disease [22]. Our data suggest that patients admitted with critical COVID-19 develop higher NAb titers and retain higher titers for at least six months after symptom onset compared to non-critically ill patients. These findings may indicate that patients with critical COVID-19 are better protected against reinfection after discharge as NAbs are strongly correlated with protection from reinfection [23, 24]. Previous studies have found a strong correlation between the levels of anti-spike Ab and disease severity [14, 25, 26]. Current studies also report similar findings regarding the association between the levels of anti-spike NAb and disease severity [9–14], with one exception [27]. These reports are however almost entirely based on pseudovirus assays. This is primarily due to live virus assays requiring BSL-3 facilities and are more time and resource-consuming. Live virus NAb assay is the most accurate method to assess antibody/virus interactions by assessing neutralization of the SARS-CoV-2 spike protein and all other parts of the SARS-CoV-2 virus [28]. This study presents NAb results based exclusively on a live virus assay, which is the method closest to describing the reality of antibody/virus interactions during SARS-CoV-2 infection [29].

The association between NAb titers and disease severity are not entirely understood, but two main explanations have been suggested. First, high disease severity could result from hyperinflammation, independent of viral load [30–32] or second, high viral load leads to increased disease severity, which then, in turn, promotes antibody production [15, 33]. However, our findings did not find associations between peak viral load during admission and disease severity, which then, in turn, would affect NAb titers. Therefore, our findings suggest that hyperinflammation is likely involved in the positive association between increasing NAb titers and disease severity.

In our study, the median time from symptom onset to first viral sample collection was seven days. Previous studies had suggested that a successful viral culturing attempt is highly dependent on samples with high viral load, where the probability was described as < 5% when the sample cycle threshold (ct) value was > 24 [34–36]. Only six of all samples collected had a ct value < 24, of which one ended up being the only successful viral culturing attempt in the study. A plausible explanation for the abundance of samples with low admission viral loads is the time between symptom onset and sample collection. In addition, viral samples were stored for a median time of 8 months at − 80 °C, which could further affect the sample viral load at the time of analysis. Our data is in line with other studies suggesting that severe and critical SARS-CoV-2 infection can be characterized as a biphasic illness with a viral replication phase and a hyperinflammatory phase [37]. Our results suggest that almost all patients were in the hyperinflammatory phase at admission. Future studies investigating SARS-CoV-2 infectiousness should focus on collecting viral samples 1–4 days after symptom onset to maximize the success rate of viral culturing attempts. Large-scale studies are needed to fully assess the risk of SARS-CoV-2 transmission at admission and further explore the clinical characteristics associated with the difference in NAb titers between disease severity groups.

In our study, none of the participants were known to be immunosuppressed. Immunosuppression is well known to affect both humoral responses after natural infection and vaccination and also the persistency of viral shedding and the neutralizing activity of antibodies, all factors that could have influenced our results in case of immunocompromised patients were included [4, 36, 38–41]. Studies focusing on antibody responses and viral shedding in immunocompromised individuals are warranted in the future.

None of the included patients in this study were vaccinated or have had a previous SARS-CoV-2 infection. Furthermore, none of the included patients were vaccinated prior to admission. We therefore assume that none of the patients included in this study were primed by a previous infection, which otherwise could affect the results.

The emergence of new dominant SARS-CoV-2 variants has been described to be associated with changes in disease severity and the effectiveness of vaccines [42, 43]. We did not have specific variant information at the individual patient level. However in Denmark, as was also the case worldwide, the wildtype-like variant containing the S:D614G mutation (formerly referred to as the Wuhan variant) was the dominant circulating variant until December 2020, where the alpha variant (B.1.1.7) quickly took over [44]. The latter dominated until June 2021. In our study, the vast majority of patients were included when the wildtype-like variant by far still was the dominating circulating variant and were therefore most likely infected with this variant. None of the patients were vaccinated at the time of inclusion, which allowed for insight into the natural humoral response and association to the severity of disease of the SARS-CoV-2 wildtype-like variant.

Evidence regarding antibody and T cell cross-reactivity between SARS-CoV-2 and the four endemic coronaviruses (NL63, 229E, OC43, and HKU1) was established during the first year of the pandemic [45–49]. Since then, studies exploring the clinical significance of antibody cross-reactivity have led to mixed results with no conclusive evidence regarding clinical outcomes [50]. In our study, we could not perform the serological analyses necessary to assess cross-reactivity with other coronaviruses besides SARS-CoV-2. Therefore, we cannot rule out the possibility that cross-reactivity was present and potentially affecting our results. Our study had no specific inclusion criteria based on previous infections. Therefore, we assume that if antibody cross-reactivity was present, it would have been randomly distributed in the study population resulting in no overall changes in our comparisons between patient groups and sample time points.

The primary strength of our study is the prospective design with sample collection at predetermined time points for up to six months after inclusion. The included patients represent the general COVID-19 population hospitalized with x-ray confirmed pneumonia during the inclusion period. Furthermore, fully validated gold standard methods were used throughout the study. However, our findings are limited by relatively low sample size, preventing the possibility of generalization. The study was also affected by missing samples. In addition, our findings were mainly from a non-vaccinated population infected primarily with the wild-type (Wuhan-Hu-1) or alpha (B.1.1.7) SARS-CoV-2 variants. Therefore, the findings will not necessarily be translatable to a vaccinated population or populations infected with a different SARS-CoV-2 variant.

## Conclusions

In conclusion, our findings support previous reports regarding the association between NAb titers and COVID-19 disease severity and contribute to new results regarding the length of the association, which was observed for up to 6 months after symptom onset. Furthermore, no SARS-CoV-2 virus was culturable seven days after symptom onset, which may have implications for infection control regimes.

## Supplementary Information

**Additional file 1: **Details regarding methods used in this study are provided in the additional file appendix.

## Data Availability

All relevant clinical data are available in the manuscript. Further data can be made available upon request.

## Abbreviations

NAb: Neutralizing antibody
COVID-19: Severe Coronavirus disease 2019
SARS-CoV-2: Severe acute respiratory syndrome coronavirus 2
Ab: Antibody
RBD: Receptor-binding domain
RT-PCR: Reverse transcriptase-polymerase chain reaction
HFNC: High-flow nasal cannula
NIV: Non-invasive mechanical ventilation
RT-qPCR: Reverse transcriptase-quantitative polymerase chain reaction
RdRp: RNA-dependent-RNA-polymerase
CPE: Cytopathogenic effect
TCID_50_: Median tissue culture infectious dose
LME: Linear mixed-effect model
MCAR: Missing completely at random
ct: Cycle threshold

## References

[1] Jiang S, Hillyer C, Du L (2020). Neutralizing antibodies against SARS-CoV-2 and other human coronaviruses. Trends Immunol.

[2] Merad M, Blish CA, Sallusto F, Iwasaki A (2022). The immunology and immunopathology of COVID-19. Science.

[3] Bonilla FA, Oettgen HC (2010). Adaptive immunity. J Allergy Clin Immunol.

[4] Chvatal-Medina M, Mendez-Cortina Y, Patiño PJ, Velilla PA, Rugeles MT (2021). Antibody responses in COVID-19: a review. Front Immunol.

[5] Pang NY, Pang AS, Chow VT, Wang DY (2021). Understanding neutralising antibodies against SARS-CoV-2 and their implications in clinical practice. Mil Med Res.

[6] Klein SL (2012). Sex influences immune responses to viruses, and efficacy of prophylaxis and treatments for viral diseases. BioEssays.

[7] Palacios-Pedrero MÁ (2021). Aging and options to halt declining immunity to virus infections. Front Immunol.

[8] Maier HE (2022). SARS-CoV-2 infection-induced immunity and the duration of viral shedding: results from a Nicaraguan household cohort study. Influenza Other Respi Viruses.

[9] Maciola AK (2022). Neutralizing antibody responses to SARS-CoV-2 in recovered COVID-19 patients are variable and correlate with disease severity and receptor-binding domain recognition. Front Immunol.

[10] Chen X (2020). Disease severity dictates SARS-CoV-2-specific neutralizing antibody responses in COVID-19. Signal Transduct Target Ther.

[11] Jeewandara C (2021). SARS-CoV-2 neutralizing antibodies in patients with varying severity of acute COVID-19 illness. Sci Reports.

[12] Hansen CB (2021). SARS-CoV-2 antibody responses are correlated to disease severity in COVID-19 convalescent individuals. J Immunol.

[13] Legros V, Denolly S, Vogrig M, Boson B, Siret E, Rigaill J, Pillet S, Grattard F, Gonzalo S, Verhoeven P, Allatif O (2021). A longitudinal study of SARS-CoV-2-infected patients reveals a high correlation between neutralizing antibodies and COVID-19 severity. Cell Molecul Immunol.

[14] Garcia-Beltran WF (2021). COVID-19-neutralizing antibodies predict disease severity and survival. Cell.

[15] Dadras O, Afsahi AM, Pashaei Z, Mojdeganlou H, Karimi A, Habibi P, Barzegary A, Fakhfouri A, Mirzapour P, Janfaza N, Dehghani S (2022). The relationship between COVID-19 viral load and disease severity: a systematic review. Immun, Inflammation Disease.

[16] Corman VM (2020). Detection of 2019 novel coronavirus (2019-nCoV) by real-time RT-PCR. Eurosurveillance.

[17] Harritshøj LH (2021). Comparison of 16 serological SARS-CoV-2 immunoassays in 16 clinical laboratories. J Clin Microbiol.

[18] Frische A (2022). Optimization and evaluation of a live virus SARS-CoV-2 neutralization assay. PLoS ONE.

[19] Lassaunière R (2021). In vitro characterization of fitness and convalescent antibody neutralization of SARS-CoV-2 cluster 5 variant emerging in mink at Danish farms. Front Microbiol.

[20] Lassaunière R, Polacek C, Gram GJ, Frische A, Tingstedt JL, Krüger M, Dorner BG, Cook A, Brown R, Orekov T, Putmon-Taylor T. Preclinical evaluation of a candidate naked plasmid DNA vaccine against SARS-CoV-2. Npj Vaccines 2021;6(1):1-3. 10.1038/s41541-021-00419-z PMC8688418 34930909

[21] Eddelbuettel D, François R (2011). Rcpp: seamless R and C++ integration. J Stat Softw.

[22] Kaygusuz, S., Korukluoğlu, G., Yasemin Coşgun, |, Şahin, | Ömer & Ferhat Arslan, |. Investigation and long-term monitoring of the presence of neutralizing antibody in patients with COVID-19 disease of different clinical severity. J Med Virol (2022) 10.1002/JMV.27751. 10.1002/jmv.27751 35365870

[23] Cromer D (2022). Neutralising antibody titres as predictors of protection against SARS-CoV-2 variants and the impact of boosting: a meta-analysis. The Lancet Microbe.

[24] Khoury DS (2021). Neutralizing antibody levels are highly predictive of immune protection from symptomatic SARS-CoV-2 infection. Nat Med.

[25] Secchi M (2020). COVID-19 survival associates with the immunoglobulin response to the SARS-CoV-2 spike receptor binding domain. J Clin Invest.

[26] Shrock E (2020). Viral epitope profiling of COVID-19 patients reveals cross-reactivity and correlates of severity. Science.

[27] Gozalbo-Rovira R, Gimenez E, Latorre V, Frances-Gomez C, Albert E, Buesa J, Marina A, Blasco ML, Signes-Costa J, Rodriguez-Diaz J, Geller R (2020). SARS-CoV-2 antibodies, serum inflammatory biomarkers and clinical severity of hospitalized COVID-19 patients. J Clinic Virol.

[28] Bewley KR (2021). Quantification of SARS-CoV-2 neutralizing antibody by wild-type plaque reduction neutralization, microneutralization and pseudotyped virus neutralization assays. Nat Protoc.

[29] Chen M, Zhang XE (2021). Construction and applications of SARS-CoV-2 pseudoviruses: a mini review. Int J Biol Sci.

[30] Gustine JN, Jones D (2021). Immunopathology of Hyperinflammation in COVID-19. Am J Pathol.

[31] Bonnet B (2021). Severe COVID-19 is characterized by the co-occurrence of moderate cytokine inflammation and severe monocyte dysregulation. EBioMedicine.

[32] Del Valle DM (2020). An inflammatory cytokine signature predicts COVID-19 severity and survival. Nat Med.

[33] Knudtzen FC (2021). SARS-CoV-2 viral load as a predictor for disease severity in outpatients and hospitalised patients with COVID-19: a prospective cohort study. PLoS ONE.

[34] Wölfel R (2020). Virological assessment of hospitalized patients with COVID-2019. Nature.

[35] Bullard J (2020). Predicting infectious severe acute respiratory syndrome coronavirus 2 from diagnostic samples. Clin Infect Dis.

[36] van Kampen JJA (2021). Duration and key determinants of infectious virus shedding in hospitalized patients with coronavirus disease-2019 (COVID-19). Nat Commun.

[37] Trougakos IP (2021). Insights to SARS-CoV-2 life cycle, pathophysiology, and rationalized treatments that target COVID-19 clinical complications. J Biomed Sci.

[38] Ryan, A. et al. Efficacy of covid-19 vaccines in immunocompromised patients: systematic review and meta-analysis. 10.1136/bmj-2021-068632. 10.1136/bmj-2021-068632 PMC8889026 35236664

[39] Haggenburg S (2022). Quantitative analysis of mRNA-1273 COVID-19 vaccination response in immunocompromised adult hematology patients. Blood Adv.

[40] Aydillo T (2020). Shedding of viable SARS-CoV-2 after immunosuppressive therapy for cancer. N Engl J Med.

[41] Org A (2021). Disease- and therapy-specific impact on humoral immune responses to COVID-19 vaccination in hematologic malignancies. Blood Cancer Discov.

[42] Esper FP (2022). Alpha to omicron: disease severity and clinical outcomes of major SARS-CoV-2 variants. J Infect Dis.

[43] Buchan SA (2022). Estimated effectiveness of COVID-19 vaccines against omicron or delta symptomatic infection and severe outcomes. JAMA Netw Open.

[44] Outbreak.info SARS-CoV-2 data explorer. https://outbreak.info/location-reports?xmin=2022-06-09&xmax=2022-12-09&loc=DNK&muts=S%3AD614G&pango=B.1.1.7&pango=B.1.177&selected=B.1.1.7&selected=B.1.177&selected=S%3AD614G.

[45] Le Bert N (2020). SARS-CoV-2-specific T cell immunity in cases of COVID-19 and SARS, and uninfected controls. Nat.

[46] Bacher P (2020). Low-avidity CD4+ T cell responses to SARS-CoV-2 in unexposed individuals and humans with severe COVID-19. Immunity.

[47] Braun J (2020). SARS-CoV-2-reactive T cells in healthy donors and patients with COVID-19. Nat.

[48] Mateus J (2020). Selective and cross-reactive SARS-CoV-2 T cell epitopes in unexposed humans. Science.

[49] Ng KW (2020). Preexisting and de novo humoral immunity to SARS-CoV-2 in humans. Science.

[50] Murray SM (2022). The impact of pre-existing cross-reactive immunity on SARS-CoV-2 infection and vaccine responses. Nat Rev Immunol.

