# Supplementary material for: High titers of neutralizing SARS-CoV-2 antibodies six months after symptom onset are associated with increased severity in COVID-19 hospitalized patients
Source: Virol J. 2023 Jan 25;20:14. doi: 10.1186/s12985-023-01974-8 (PMC9875770; doi:10.1186/s12985-023-01974-8)
Supplement: Supplementary file 1 — Additional file 1: Details regarding methods used in this study are provided in the additional file appendix. [file 12985_2023_1974_MOESM1_ESM.docx]

**Supplemental Methods**

**Nucleic acid extraction and quantitative real-time reverse-transcriptase polymerase chain reation (RT-qPCR) for SARS-CoV-2**

Collected viral samples were contained in 2 ml universal transport medium (UTM) and stored in -80°C. SARS-CoV-2 RNA was purified from 200 µl patient samples (extracted from UTM) on a Magna Pure 24 instrument using the MagNA Pure 24 Total NA Isolation Kit (Roche Molecular Systems, Inc.) with a final elution volume of 100 µl.

Detection of SARS-CoV-2 RNA was performed using E-Sarbeco RT-PCR as described by the authors^1^. In order to quantify the viral load of the samples, calibration curves using serial dilutions of a Synthetic SARS-CoV-2 RNA control (10^6^ RNA copies/µl) (Twist Bioscience, CA, USA) were performed at four independent runs prior to the experiment on which a formula was based to calculate viral load from the Ct values detected in the patient sample. In every PCR setup was included two intermediat dilutions of the calibration curve to ensure PCR quality. Formula used for calculation of viral load from ct value (x) was (-0,293x + 13,145)+1 (to account for dilution).

**Viral culturing**

Oropharyngeal swab samples in UTM were pretreated by sterile filtration through 0,45 µM polyethersulfone microfilter. Monolayers with 90 % confluent African green monkey kidney cells (VERO-E6) in the exponential growth phase were prepared in 48-well TC-treated plates. Growth medium consisted of Eagles minimum essential medium with 1% fetal calf serum, 1 % penicillin-streptomycin and 1 % L-Glutamin. Before inoculation, cell-preparation medium was removed and 100 µL of fresh growth medium was added to each well. The sterile-filtered samples was added to three wells per sample in a volume of 100 µL per well. The plates were incubated for one hour at 37ºC and 5% CO_2_ before further addition of 800 µL growth medium. The plates were incubated at 37ºC and 5% CO_2_ for 3-4 days. The plates were inspected daily in microscope for cytopathogenic effect (CPE). After 3-4 days the first passage was harvested by cell scraping and the supernatant was passaged in new plates. If the second passage showed clear CPE the virus isolate was harvested and the positive result was confirmed by qRT-PCR. If CPE was absent after second passage a third passage was performed. If CPE still was absent after third passage, the sample was considered negative for infectious virus.

**Variable Definitions**

Variables included: comorbidities which were used to calculate the Charlson Comorbidity Index, immunosuppression, vital signs at admission used for calculation of Early Warning Score, time from symptom onset to admission, peak supplementary oxygen treatment during admission, pharmacological treatment, admission length and death within 180 days of inclusion. Immunocompromised status was defined as; hematological malignancies, AIDS (HIV with CD4 <200 cells/µl), treatment with stem cell or organ transplantations, and other immunosuppressive treatments prior to admission (details in supplementary appendix). Immunosuppressive treatment was defined as use of: (1) corticosteroid treatment exceeding a prednisolone-equivalent dose of 20 mg daily > 14 days at the time of admission, (2) monoclonal antibodies interfering with the immune system, (3) small molecular immunosuppressants or (4) antineoplastic agents.
For optimal description of clinical status upon admission, the Early Warning Score (EWS) with the highest volume per minute of supplemental oxygen within the first 24 hours of admission was chosen as the admission EWS score.

**TCID_50_ microneutralization assay**

Heat inactivated samples were 2-fold serial diluted and inoculated with 300 x TCID_50_ of SARS-CoV-2 early pandemic strain (lineage B.1) at 37°C, 5% CO_2_ for 1 hour to allow neutralization. 100 µl of each sample/ virus dilution were transferred to a 96 well tissue culture plate seeded with 10^4^ Vero E6 cells the preceding day, and incubated for 24 hours at 37°C, 5% CO_2_. Infection medium was removed, and the plates were washed twice with 100 µl of PBS and fixed with 100 µl of 80 % (v/v) acetone in PBS for 10 minutes. Fixation was removed and the plates were left to air dry. Plates were washed 3 times with a 30 second soak in wash buffer (PBS containing 1% (v/v) Triton-X100) and 100 µl of diluted anti SARS-CoV-2 nucleocapsid protein mouse monoclonal antibody clone 7E1B (1:4000 dilution; Cat. # BSM-41414M, Bioss, Woburn, Massachusetts, USA) was added to each well. Plates were incubated for 5 minutes on an orbital shaker (300 rpm) at room temperature and afterwards for 1 hour at 37°C. Plates were washed 3 times as described above followed by an addition of 100 µl of 1:10000 diluted goat anti-mouse IgG (H+L) cross-adsorbed HRP conjugate antibody (Cat. # A16078; Invitrogen, Waltham, Massachusetts, USA) to each well. Plates were incubated for 5 minutes on an orbital shaker (300 rpm) at room temperature and afterwards for 1 hour at 37°C. Plates were washed 5 times as described above and subsequently washed 3 times in deionized water with no soak. 100 µl of TMB One Substrate (Cat. # 4380, KemEnTec, Denmark) was added to each well and the plates were incubated for 15 minutes at room temperature in the dark. 100 µl of sulfuric acid was added to each well to stop the reaction. Absorbance was read at 450 nm with 620 nm as a reference on a FLUOstar Microplate Reader (BMG LABTECH, Germany). A 50 % cut-off value was calculated from quadruplicate virus and cell control wells included on each plate using the following equation: (average optical density (OD) of virus control wells + average OD of cell control wells)/2. The 50 % neutralization titer was calculated as the interception between the cut-off value with a four-parameter logistic regression curve fitted for each serum serial dilution and reported as the reciprocal value of the last sample dilution giving a 50 % neutralization of virus. To minimize inter-assay variation, the titers were normalized according to a positive control included on each assay plate.

1. Corman VM, Landt O, Kaiser M, et al. Detection of 2019 novel coronavirus (2019-nCoV) by real-time RT-PCR. *Eurosurveillance*. 2020;25(3):2000045. doi:10.2807/1560-7917.ES.2020.25.3.2000045/CITE/PLAINTEXT
